# Is There Any Association Between Chronic Periodontitis and Anxiety in Adults? A Systematic Review

**DOI:** 10.3389/fpsyt.2021.710606

**Published:** 2021-08-03

**Authors:** Walessa Alana Bragança Aragão, de Deiweson Souza-Monteiro, Deborah Ribeiro Frazão, Yago Gecy de Sousa Né, Railson de Oliveira Ferreira, Luis Felipe Sarmiento Rivera, Miki Taketomi Saito, Cassiano Kuchenbecker Rösing, Nathalia Carolina Fernandes Fagundes, Lucianne Cople Maia, Rafael Rodrigues Lima

**Affiliations:** ^1^Laboratory of Functional and Structural Biology, Institute of Biological Sciences, Federal University of Pará, Belém, Brazil; ^2^Laboratory of Neuroscience and Behavior, Federal University of Pará, Belém-Pará, Brazil; ^3^School of Dentistry, Institute of Health Sciences, Federal University of Pará, Belém, Brazil; ^4^Department of Periodontology, Faculty of Dentistry, Federal University of Rio Grande Do Sul, Porto Alegre, Brazil; ^5^School of Dentistry, Faculty of Medicine and Dentistry, University of Alberta, Edmonton, AL, Canada; ^6^Department of Pediatric Dentistry and Orthodontics, School of Dentistry, Federal University of Rio de Janeiro, Rio de Janeiro, Brazil

**Keywords:** periodontitis, periodontal disease, anxiety, oral health, emotional disorders, systematic review

## Abstract

Periodontitis is a multifactorial disease triggered by dysbiotic biofilms, involving the host's immune response, systemic and behavioral factors, including psychosocial conditions. This systematic review aimed to investigate the possible association between periodontitis and anxiety in adults. Searches were performed in PubMed, Scopus, Web of Science, Lilacs, Cochrane, and OpenGrey databases, without language restrictions, considering studies in adults (P-Participants), with (E-Exposure) and without periodontitis (C- Comparison) in an outcome of association with anxiety (O-outcome). Methodological quality assessment was carried out using the Newcastle-Ottawa protocol for case-control and cross-sectional studies, followed by an analysis of the level of evidence using the GRADE tool. Metanalysis was not performed due to several differences in methods applied by authors in primary studies. Eleven observational studies were selected according to the inclusion criteria from the total of 6,380 studies retrieved from databases. Eight studies demonstrated higher anxiety levels in subjects with periodontitis, among which only one study presented a high risk of bias. The GRADE tool revealed a low level of evidence for the anxiety outcome measured by the State-Trait Anxiety Inventory (STAI), both for case-control and cross-sectional studies. However, since anxiety may affect the quality of life of many subjects, it reinforces the need for further studies that evaluate this association for more extended periods.

**Clinical Trial Registration:**PROSPERO-CRD42020190445.

## Introduction

Periodontitis is one of the most prevalent diseases in the oral cavity that develops as a chronic inflammatory process in response to a dysbiotic biofilm on the surfaces of teeth (1). Its etiology is multifactorial, and dysbiotic dental biofilms play a significant role in the initiation and progression of the disease (2). Thus, this disease is characterized by progressive destruction of the supporting tissues of the teeth, i.e., periodontal ligament and alveolar bone, and may lead to tooth loss if left untreated (3, 4).

The host's inflammatory and defensive responses that lead to bone loss may suffer influence from genetics and environmental factors (5). Systemic factors, such as diabetes mellitus (6) and smoking (7), can contribute as modifying factors to the progression of periodontitis by increasing the release of pro-inflammatory mediators, such as interleukins (IL-1α, IL-1β and IL-6), tumor necrosis factor-alpha (TNF-α) and prostaglandins (8, 9).

In this context, psychological stress may be associated with systemic inflammatory processes that, in turn, can contribute to the progression and/or worsening of periodontitis (10). Among the emotional disorders that generate psychological stress, anxiety is currently the most common and present a prevalence of 3.6%, affecting a large part of the world population in the most several social, occupational, family, and individual configurations, which reflects the dynamics of modern society in the context of daily stress (11).

Anxiety affects cognition, decision-making, and performance of various individuals' daily activities (12–14). Furthermore, anxiety can influence processes associated with blood pressure control (15) and the exacerbation of inflammatory reactions resulting from the state of psychological stress (16, 17). Thus, it can be considered that individuals with systemic and oral pathologies may present a worsening of the prognosis through high levels of anxiety (18).

Previous studies have indicated a possible association between periodontitis and psychological stress (19–22). Considering this evidence, it can be understood that stress is one of the events exacerbated in individuals with anxiety disorder, where one of the main physiological interaction mechanisms to consider is the increase in the inflammatory load and cortisol levels in the blood (23, 24). This physiological interaction constitutes a relevant measure in assessing the risk of periodontitis as demonstrated in other systematic reviews (25–27). Despite this evidence, the association between periodontitis and anxiety still needs to be elucidated. Therefore, this review aimed to identify an association between periodontitis and anxiety and suggest the possible mechanisms involved in this process.

## Materials and Methods

### Protocol and Registration

This systematic review was recorded in Prospective Register of Systematic Review (PROSPERO-CRD42020190445) and conducted based on the Preferred Reporting Items for Systematic Reviews and Meta-Analysis (PRISMA) guidelines (28).

### Eligibility Criteria

The criteria were defined according to the PECO strategy considering observational studies with adult humans (Participants) with periodontitis (Exposure) and without periodontitis (Comparison) and its association with the presence of anxiety (Outcome). The focused question of this review is this: “Is chronic periodontitis associated with anxiety in adults?”

The inclusion criteria considered was observational studies with individuals with chronic periodontitis and individuals with no history of the disease in which data from anxiety levels was evaluated. Studies including adults with neurological or cognitive disorders were excluded. Case reports, reviews, descriptive studies, opinion articles, technical articles, animal studies, and *in vitro* studies were also excluded.

### Study Search and Selection Strategy

The following online databases were accessed: PubMed, Lilacs, Scopus, Web of Science, Cochrane, and Open Gray, from October 2020 to December 2020. No restrictions were applied to searches regarding publication date or language.

The search strategies were adapted according to each database (Supplementary Table 1), in which a research alert was created to notify new studies according to the described search strategy.

Relevant citations were saved in a reference manager (EndNote, version X9, Thomson Reuters). First, duplicate results were removed. Then, titles and abstracts were analyzed according to the inclusion and exclusion criteria. Finally, the remaining articles were evaluated based on the full-text reading. Additional citations were sought from the reference list of all previously selected articles. The selection process was conducted independently by two examiners (W.A.B.A and D.S.M.) and verified by a third examiner (R.R.L.) in cases of disagreement.

### Data Extraction

The eligible articles were extracted and tabulated by two examiners (W.A.B.A. and D.S.M.) and verified by a third examiner (Y.G.S.N.). The following data were recorded: author/year, type of study, participants (sample source, sample size, age, and characteristics of groups), clinical parameters (periodontal evaluation, anxiety evaluation), statistical analysis, and results. In the absence of information about the studies, the authors were contacted by e-mail for up to 4 consecutive weeks.

### Risk of Bias

Two examiners (W.A.B.A. and D.R.F.) independently assessed the methodological quality and risk of bias by the Newcastle-Ottawa protocol for case-control studies (29) and a protocol adapted for cross-sectional studies (30). A third examiner was consulted (R.O.F.) in case of doubts about the evaluation in any criterion.

The protocol for case-control studies consists of domains that analyze the representativeness of cases and definition of controls, group comparability, determination of exposure, and non-response rate. For cross-sectional studies, the protocol covers the representativeness and size of the samples, non-responders, comparability, evaluation of the result, and statistical test. The examiners standardized the checklist to provide valid information and the viability of the methods. Thus, an asterisk (^*^) was applied according to the agreement of the questions in each domain, and a dash (-) was used when the topic analysis was not applicable. The criteria used for the quality assessment are available in Supplementary Tables 2, 3.

### Level of Evidence

The selected studies were grouped to analyze the level of evidence of the outcome using the “Grading of recommendations, assessment, development and evaluation” (GRADE) tool, as the narrative evidence profile, which was evaluated using criteria such as study design, risk of bias, inconsistency, indirectness and imprecision (31). As all studies are observational, the level of evidence starts low and, it can be reduced during the analysis if there is a serious or very serious problem, or it can be increased depending on the dose-effect, confounding factors, and the magnitude of effect.

## Results

### Selected Studies

**Figure 1:**
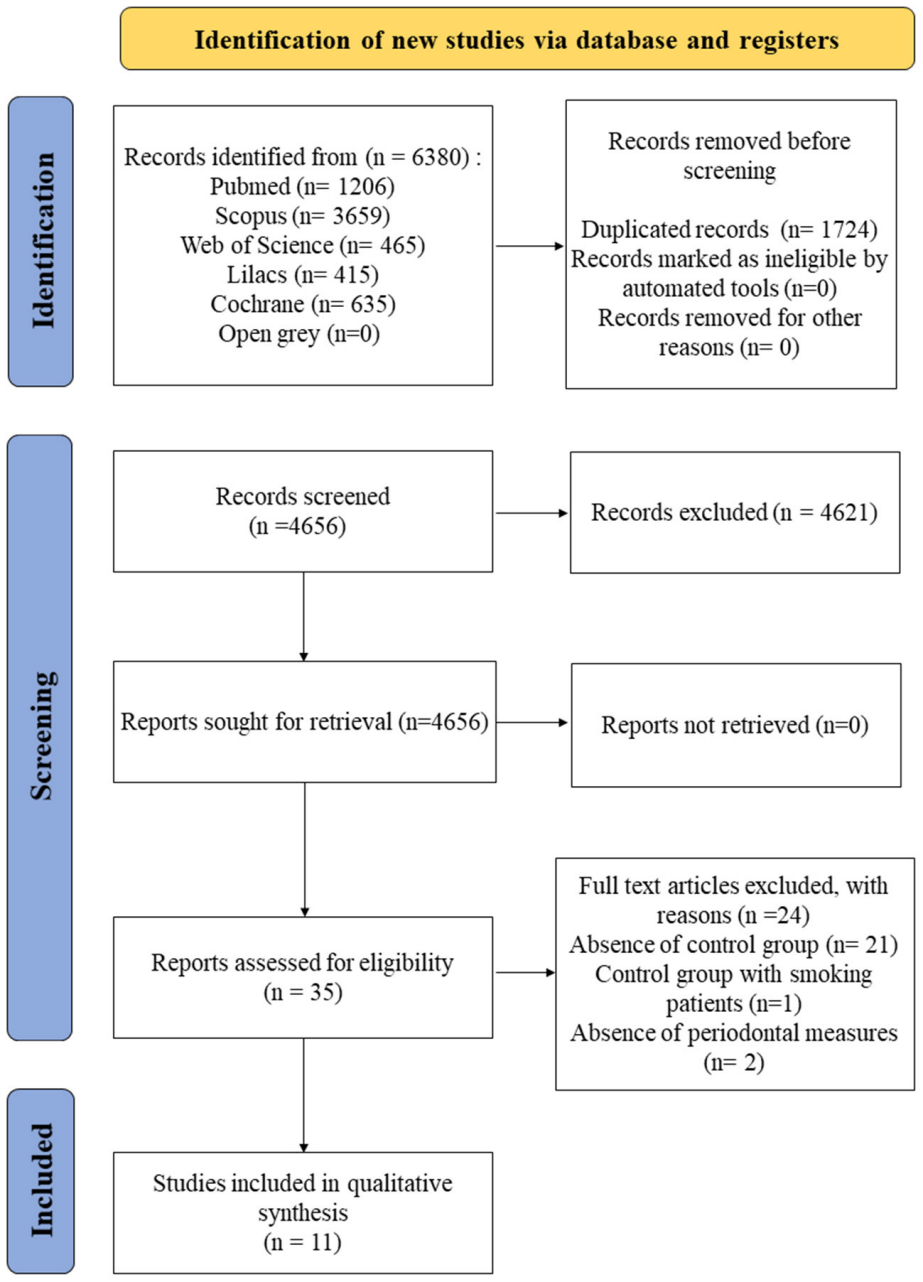
Flowchart of selection of studies according to the PRISMA guidelines.

The total number of studies found in the databases was 6,380, based on the search strategy defined by the PECO. From these, 1,724 duplicates were excluded; and 4,621 were removed by title and abstract analysis. Therefore, 35 studies were selected for full-text reading. The methodological characteristics of the studies were observed as study design, considering the inclusion and exclusion criteria, and 24 studies were excluded due to the presence of control group with smokers (32), absence of periodontal measures (33, 34), and lack of a control group (35–55). Therefore, after full-text reading, 11 studies were selected for qualitative analysis (10, 56–65). Figure 1 describes the flowchart of the studies selection according to the PRISMA protocol. No quantitative analysis was performed since the nature and heterogeneity of the studies was high, not making merging of data possible.

### Characteristics of the Studies

All selected studies were observational—six were case-control (10, 56, 59–61, 63), and five were cross-sectional design (57, 58, 62, 64, 65). Studies were performed in several countries, including Brazil (10, 56, 57, 59), Italy (62), Austria (58), Jordan (60), China (61), Israel (63), India (64), and Iran (65). The number of participants involved in each study ranged from 46 to 300 patients. The age of the participants ranged from 14 to 71 years old.

Included studies present a control group without periodontitis and a case group with diagnosed periodontitis through periodontal measurements of several parameters such as clinical attachment level (CAL), plaque index (PI), probing pocket depth (PPD), bleeding on probing (BOP), plaque control record (PCR), gingival index (GI), probing depth (PD), dental mobility (DM), furcations (F), calculus index (CI), papillary bleeding index (PBI) and approximal plaque index (API).

For studies that did not provide the odds ratio (OR) data with a 95% confidence interval (CI), we performed the calculation using the GraphPad Prism 7.0 software (GraphPad Software Inc., La Jolla, CA, USA).

In the study by Vettore et al. (56), two calibrated examiners evaluated CAL, PPD, GI, and PI at six sites per tooth (mesiobuccal, buccal, distobuccal, distolingual, lingual, and mesiolingual) at all teeth excluding third molars. Participants were allocated into three groups according to their PPD levels: control (<4 sites with PPD = 4.0 mm), test group 1 (at least four sites with PPD ≥ 4 mm and ≤ 6 mm), and test group 2 (at least four areas with PPD > 6 mm). Anxiety assessment was performed using the State-Trait Anxiety Inventory (STAI) method. This is one of the most used methods to assess anxiety levels consisting of two self-report subscales that determine how the subject generally feels (T-trace) and how he feels at a given moment (S-state) (66). According to the Non-parametric Spearman linear correlation, moderate CAL frequencies (*p* < 0.05), moderate PPD frequencies (*p* < 0.05), and PPD frequencies >4 mm (*p* < 0.05) were found to be significantly associated with high scores of anxiety trait.

Solis et al. (57) defined study cases as subjects with two or more interproximal sites from different teeth with a CAL of ≥ 6 mm and at least one additional site with a pocket depth (PD) of ≥ 5 mm. Subjects that did not meet this criterion were allocated to the control group. After defining the groups, a calibrated examiner evaluated CAL, PPD, GI, and PI, and the subjects answered the STAI questionnaire. There were no significant differences between groups (*p* = 0.57; Mann–Whitney test). Thus, the findings of this study do not confirm the hypothesis of an association between periodontitis and anxiety.

In the study of Laforgia et al. (62), an examiner evaluated CAL, PPD, GI, DM, F, and PCR. Anxiety was assessed using the Symptom Check List (SCL-90), which consists of a questionnaire composed of 90 symptoms and nine symptom dimensions: somatization, obsessive-compulsive disorder, interpersonal sensitivity, phobic anxiety, paranoid ideation, psychoticism, hostility, depression, and anxiety (67). The percentage of anxiety was higher in the group with periodontitis (31.48%) than the control (20.37%), implying that the hypothesis of a correlation between periodontal and psychological variables can be confirmed. Thus, to support that information, the linear regression analysis showed a positive correlation between periodontal conditions and SCL-90 scores.

Saletu et al. (58) diagnosed periodontitis based on clinically and radiographically evident loss of attachment. Three groups were distinguished considering the severity of periodontitis: slight periodontitis (CAL of 1–2 mm and/or a bone loss of 10–30%), moderate periodontitis (CAL of up to 4 mm, and/or a bone loss of 30–50%), and severe periodontitis (CAL of ≥ 5 mm and/or a bone loss of >50%). Participants in the control group had no history of the disease and showed no bone loss and no probing depth >4 mm. Subjects with gingivitis were allocated to the control group. Anxiety assessment was performed using the Self-Rating Anxiety Scale (SAS), which consists of 20 items that include questions about psychological and physical symptoms related to anxiety from positive and negative perspectives (68, 69). Responses range from 1 (none/little) to 4 (all the time), and scores that are equal to or exceed a specified cutoff point are an indication of the likely presence of anxiety (69). This study showed that anxiety scores were higher in subjects with periodontitis (Multifactorial analysis of variance; Case: 31.1 ± 10.1; Control: 25.3 ± 4.0; *p* = 0.018) in addition to a positive correlation of anxiety scores with the severity of periodontitis and CAL levels, under the control of covariates such as age, smoking, and API (Partial correlation; *p* ≤ 0.05).

In the publication of Vettore et al. (59), participants were divided into three groups based on PPD levels: control (<4 sites with PPD ≤ 4.0 mm; *n* =20), test group 1 (at least four sites with PPD ≥ 4.0 and ≤ 6.0 mm; *n* =26), and test group 2 (at least four areas with PPD > 6 mm; *n* = 20). Anxiety was assessed using the STAI method. Two calibrated examiners assessed CAL, PPD, PI, Gingival Index (GI) at six sites per tooth (mesiobuccal, buccal, distobuccal, distolingual, lingual, and mesiolingual) at all teeth, except third molars. Non-parametric Spearman linear correlation showed a significant correlation between anxiety trait and PPD (*p* < 0.05) and CAL (*p* < 0.05).

Castro et al. (10) diagnosed periodontitis considering a CAL ≥ 4 mm and BOP in at least 10 teeth and probing pocket depth PPD ≥ 6 mm in at least five teeth. A single examiner assessed CAL, PPD, and BOP in both study groups. Anxiety was evaluated using the STAI and Beck Anxiety Inventory (BAI) methods. The latter consists of a self-report scale consisting of 21 items or descriptive statements of anxiety symptoms rated by the subject on a four-point scale. The total score allows a classification of anxiety into levels of intensity (minimum, moderate, and severe) (70, 71). The findings showed that there was no significant association between periodontitis and anxiety (STAI-T: Odds Ratio (OR) = 1.003, Confidence Interval (CI) 0.95–1.06, *p* = 0.912; BAI: OR = 0.946, CI 0.86–1.04, *p* = 0.257) in the multivariate analysis.

In the study by Ababneh et al. (60), participants were divided into three groups: control, aggressive periodontitis (AP), and chronic periodontitis (CP). The diagnosis of AP considered a CAL > 2 mm in at least two teeth, “arch-shaped” bone loss, and age <45 years. The CP group was composed of subjects with the same characteristics but older than 45 years old. Therefore, we considered for this review only the CP group to maintain the segmentation of the senior periodontitis classification, on which the studies in this review are based. Anxiety was assessed using the Hospital Anxiety and Depression (HAD) scale consisting of 14 statements (seven for anxiety and seven for depression) (72), and the periodontal evaluation included only CAL and PI. The results showed no association between periodontitis and anxiety when comparing the group with chronic periodontitis and control (*p* = 0.49; Chi-square test). Based on the data from the study, we calculated the OR (HAD scale: OR = 1.29, CI: 0.63 to 2.706; *p* = 0.57; Fisher's exact test).

Li et al. (61) evaluated periodontitis using CAL, PPD, and BOP and anxiety using the SAS method. Subjects with periodontitis were divided into three subgroups: moderate, high, severe. Based on the analysis of variance, anxiety indexes of the periodontitis group were higher than those of the control (*p* < 0.01).

Levin et al. (63) evaluated BOP, PPD, and PI in all participants, and anxiety was measured using the Dental Anxiety Scale (DAS) method. Subjects answered a questionnaire that addressed four dental scenarios for assigning up to five points in each scale's domain (73). From this, it was shown that the levels of dental anxiety were higher in subjects with periodontitis, who were more likely to fear the noise of dental instruments and the application of anesthesia (63). There was a higher percentage of anxiety in subjects with periodontitis (*p* = 0.036; Chi-square test). Based on the data from the study, we calculated the OR (Self-assessed dental anxiety: OR: 13.16; CI: 5.361–32.5; *p* < 0.0001; Fisher's exact test).

In the study by Pal et al. (64), CAL and PPD were evaluated, and subjects with periodontitis were categorized into three groups according to the American Academy of Periodontology (AAP): mild (CAL: 1–2 mm), moderate (CAL: 3–4 mm), and severe (CAL ≥ 5 mm). Anxiety was assessed using the BAI method. The findings showed an association between anxiety and the severity of periodontitis (*p* < 0.001; Chi-square test). Based on the data from the study, we calculated the OR (OR: 7.861; CI: 4.189–14.76; *p* < 0.0001; Fisher's exact test).

Naghsh et al. (65) study diagnosed periodontitis by considering a CAL ≥ 4 mm or PPD of ≥5 mm. The severity of periodontitis was categorized according to the AAP. BOP and PI were also evaluated in the subjects. Anxiety was assessed using the STAI method. A positive correlation was observed in the Pearson correlation tests between PPD and anxiety score (*p* < 0.001).

Among all these studies, eight showed a significant association between periodontitis and anxiety (56, 58, 59, 61–65) and three studies did not show significant results for this association (10, 57, 60). However, the different types of methods for assessing anxiety made it impossible to carry out a meta-analysis. The summary of the study characteristics is described in Table 1.

**Table 1:** Characteristics of the included studies.

**Author (year), study design**	**Participants**				**Clinical parameters**		**Statistical analysis**	**Results**
	**Source of sample**	**Sample size**	**Age**	**Characteristics of groups**	**Periodontal evaluation**	**Anxiety evaluation**		
Vettore et al. (56) Case-control	Rio de Janeiro, Brazil.	*n* = 79 22 control group 57 case group: *n* = 27 (moderate periodontitis), *n* = 30 (severe periodontitis)	Mean: 46.8 ± 8 years	Control: PPD ≤ 3 mm Case: moderate periodontitis: PPD ≥ 4 mm and ≤ 6 mm; severe periodontitis: PPD > 6 mm.	CAL PPD GI PI	STAI	Comparison between groups: Chi-Square and Kruskal–Wallis tests. Associations with outcome: Spearman linear correlation coefficients.	The frequency of moderate CAL and moderate PPD were found to be significantly higher in patients with more anxiety scores (*p* ≤ 0.05).
Solis et al. (57) Cross-sectional	Vale do Paraíba, Brazil	*n* =153 106 control group 47 periodontitis group	Mean: Control (34.92 ± 10.21); Case (42.91 ± 10.45)	Control: periodontally healthy subjects Case: CAL ≥ 6 mm and PPD ≥ 5 mm	CAL PPD GI PI	STAI	Comparison between groups: Mann–Whitney test	There was no difference in scale score means between patients with and without periodontitis (*p* = 0.57).
Laforgia et al. (62) Cross-sectional	Bari, Italy	*n* = 108 54 control group 54 periodontitis group	24–67	Periodontal criteria not described Control: periodontally healthy subjects Case: subjects with periodontitis	CAL PPD GI DM F PCR	SCL-90	Comparison between groups: *t*-Student test Association with outcome: linear regression model	Significantly difference in percentage of subjects with anxiety in the case group (31.48%) against control group (20.37%).
Saletu et al. (58) Cross-sectional	Vienna, Austria	*n* =81 41 control group 40 periodontitis group	Control: 23–70 Case: 32–64	Control: periodontally healthy subjects Case: CAL ≥ 4 mm	CAL PPD API PBI	SAS	Comparison between groups: Multifactorial analysis of variance Partial correlation	The mean of the anxiety scores of the group with periodontitis was higher compared to the control group (*p* = 0.018).
Vettore et al. (59) Case-control	Rio de Janeiro, Brazil.	*n* = 66 20 control group 26 test group 1 20 test group 2	Mean: 46.1 ± 8 years	Control: PPD ≤ 4 mm Test group 1: PPD ≥ 4 and ≤ 6 mm Test group 2: PPD > 6 mm	CAL PPD GI PI	STAI	Comparison between groups: Kruskal–Wallis tests. Non-parametric Spearman's linear correlation	High levels of PPD and CAL were associated with high anxiety scores before periodontal treatment (*p* ≤ 0.05).
Castro et al. (10) Case-control	Porto Alegre, Brazil	*n* = 165 69 control group 96 case group	35–60	Control: 20 teeth with CAL or PPD ≤ 3 mm Case: CAL ≥ 4 mm and BOP in at least 10 teeth, and PPD ≥ 6 mm in at least five teeth	CAL PPD BOP	BAI STAI	Comparison between groups: *t*-Student test Associations with outcome: multivariate logistic regression	There was no significant association between periodontitis and anxiety (STAI-T: OR = 1.003, CI 0.95–1.06, *p* = 0.912; BAI: OR = 0.946, CI 0.86 to 1.04, *p* = 0.257).
Ababneh et al. (60) Case-control	Jordan	*n* = 181 81 control group 100 case group	Mean: 31.3 ± 11.4	Control: periodontally healthy subjects Case: subjects with periodontitis (CAL ≥ 2 mm and PD ≥ 3 mm)	CAL PI	HAD Scale	Comparison between groups: Chi-square test	Periodontitis was not associated with anxiety. Based on the data, it was calculated the OR (1.29) and 95% CI (0.63 to 2.706), *p* = 0.57
Li et al. (61) Case-control	Changsh, China	*n* = 46 29 control group 17 case group	–	Control: periodontally healthy subjects Case: subjects with periodontitis (CAL ≥ 4 mm)	CAL PPD BOP	SAS	Comparison between groups: analysis of variance (ANOVA)	Anxiety indexes of the periodontitis group were higher than those of the control (*p* <0.01).
Levin et al. (63) Case-control	Tel-Hashome, Israel	*n* = 150 50 control group 100 case group	18–65	Control: periodontally healthy subjects Case: subjects with periodontitis (CAL ≥ 2 mm and PD ≥ 3 mm)	BOP PPD PI	Corah's DAS	Comparison between groups: Chi-square and *t*-Student tests	Higher percentage of anxiety in subjects with periodontitis (*p* = 0.036). Based on the data, it was calculated the OR (13.16) and 95% CI (5.361 to 32.5); *p* <0.0001
Pal et al. (64) Cross sectional	India	*n* = 300 150 control group 150 case group	21–71	Control: PPD ≤ 3 mm Case: PPD ≥ 4 mm in each quadrant	CAL PPD	BAI	Chi-square test	Association of anxiety and the severity of periodontitis was found to be significant (*p* <0.001). Based on the data, it was calculated the OR (7.861) and 95% CI (4.189 to 14.76); *p* <0.0001
Naghsh et al. (65) Cross sectional	Isfahan, Irã	*n* = 90 45 control group 45 case group	20–55	Control: periodontally healthy subjects Case: two areas with CAL ≥4 mm or PPD of ≥5 mm	BOP PPD PI	STAI	Comparison between groups: *t*-Student test Pearson correlation tests	Correlation direct and meaningful between PPD and anxiety score (*r* = 0.369 *P* <0.001)

*API, approximal plaque index; BAI, Beck Anxiety Inventory; BOP, bleeding on probing; CAL, Clinical attachment level; CI, confidence interval; DAS, Dental Anxiety Scale; DM, Dental mobility; F, Furcations; GI, Gingival index; HAD, Hospital Anxiety and Depression; OR, odds ratio; PBI, Papillary bleeding index; PCR, Plaque Control Record; PI, Plaque index; PPD, Probing pocket depth; SAS, Self-Rating Anxiety Scale; SCL-90, Symptom Check List; STAI, State-Trait Anxiety Inventory*.

### Risk of Bias

Analysis of methodological quality (Table 2) was carried out for case-control and cross-sectional studies. Some studies have methodological flaws regarding the lack of description of the selection process of the control group (56) and undefined controls (56, 59, 60). Additionally, the absence of description of the rate of non-respondents was observed in all studies (10, 56–65).

**Table 2:** Summary of the methodological quality analysis according to the Newcastle-Ottawa protocol to assess the risk of bias of the cross-sectional and case-control studies.

**Case-control**			
**Studies**	**Selection**	**Comparability**	**Exposure/outcomes**
Ababneh et al. (60)	***	**	**
Castro et al. (10)	****	**	**
Levin et al. (63)	***	**	**
Li et al. (61)	****	*	**
Vettore et al. (56)	**	**	**
Vettore et al. (59)	***	**	**
**Cross-sectional**			
Laforgia et al. (62)	***	-	**
Naghsh et al. (65)	***	**	**
Pal et al. (64)	***	**	**
Solis et al. (57)	***	**	**
Saletu et al. (58)	***	**	**

Case-control study: Selection- 1) Is the case definition adequate? *; 2) Representativeness of the Cases *; 3) Selection of controls *; 4) Definition of controls *; Comparability (maximum **); Exposure- 1) Determination of Exposure *; 2) Same verification method for cases and controls *; 3) Non-Response Rate *. Cross-sectional study: Selection- 1) Representativeness of the samples *; 2) Sample size *; 3) Non-respondents *; Comparability (maximum **); Result- 1) Evaluation of the result * or **; 2) Statistical test *.

Furthermore, Li et al. (61) did not control all confounding factors such as the age and sex of individuals, and Laforgia et al. (62) did not describe the possible confounding factors of the research.

No cross-sectional studies showed any justification for the sample size of the groups evaluated (57, 58, 62, 64, 65).

However, in general, most articles fulfill the criteria of the Newcastle-Ottawa protocol, namely: adequate case definition, the definition of controls, comparability, and evaluation of results. Except for the Vettore et al. (56) study, that presents problems in the control group due to lack of description, and Laforgia et al. (62) that does not describe the justification of the sample size, rate of non-respondents, and control of confounding factors.

### Level of Evidence

The narrative GRADE analysis of evidence profile included only one tool for anxiety evaluation, the State-Trait Anxiety Inventory (STAI). There were other anxiety tests in common between the articles, as the Self-Rating Anxiety Scale (SAS) and the Beck Anxiety Inventory (BAI), but the type of the studies that evaluated them was different (one was a case-control and the other was cross-sectional), which made it impossible to gather the data. Then, the certainty of evidence for the STAI test was divided into case-control (10, 56, 59) and cross-sectional studies (57, 65), and both outcomes presented a very low certainty of evidence. Serious issues with inconsistency were observed due to the lack of homogeneity in statistical evaluation among the studies and imprecision due to the magnitude of effect based on the small number of events (Table 3).

**Table 3:** Narrative evidence profile according to the Grading of Recommendation, Assessment, Development, and Evaluation (GRADE) instrument.

**Certainty assessment**							**Impact**	**Certainty**	**Importance**
**No. of studies**	**Study design**	**Risk of bias**	**Inconsistency**	**Indirectness**	**Imprecision**	**Other considerations**			
**State-Trait Anxiety Inventory (case-control studies)**									
3	observational studies	not serious	not serious	not serious	serious^b^	none	Two studies demonstrated that state-trait anxiety levels were higher on the periodontitis group (*p* < 0.05). However, the other study reported no significant association between periodontitis and anxiety (*p* = 0.257).	⊕○○○VERY LOW	IMPORTANT^c^
**State-Trait Anxiety Inventory (cross-sectional studies)**									
2	observational studies	not serious	serious^a^	not serious	serious^b^	none	One study did not find any difference in scale score means between patients with and without periodontitis (*p* = 0.57). The other study reported a direct and meaningful correlation between probing pocket depth and anxiety score (*r* = 0.369; *p* < 0.001).	⊕○○○VERY LOW	IMPORTANT^c^

a *The statistical evaluation of the studies was different, which reported opposite results*.

b *The magnitude of effect, based on a small number of events, was very low*.

c *Despite the low certainty, the evidence was considered important for influencing patients' quality of life*.

## Discussion

This systematic review showed that an increased level of anxiety was found in subjects with periodontitis compared with periodontally healthy individuals. This association was conducted in 8 studies included in this review (56, 58, 59, 61–65). Thus, psychosocial factors, such as anxiety, contribute to the development or progression of periodontitis (74).

In this context, emotional disorders can reduce the body's immune response, and increase the susceptibility to infections. This alteration may be associated with the hypothalamic-pituitary-adrenal (HPA) axis and higher cortisol levels, which have an inhibitory effect on the immune system (75). Cortisol is a glucocorticoid hormone released by the adrenal glands that, in chronic stress situations, is maintained at high levels, reducing the secretion of immunoglobulin A and G (IgA and IgG), which are essential against microbial pathogens (76). Moreover, the excess of cortisol can cause an increase in pro-inflammatory cytokines such as TNF-α, IL-6, and C-reactive protein (5, 77). On the other hand, previous evidence indicated a process of HPA axis exhaustion after prolonged periods of stress, this would lead to a reduction in the release of cortisol by the adrenal glands (78, 79), as was the case identified in a study in which anxious individuals had reduced levels of cortisol when compared to non-anxious individuals (80). Therefore, more studies must be performed to establish cortisol as an anxiety biomarker (81). Another mechanism resulting from anxiety is the stimulation of the central nervous system with the release of adrenaline and norepinephrine, which also have immunosuppressive action (82). This increased release of cortisol can shift the inflammatory response from a protective to a destructive pattern.

Thus, immune system alterations may allow increased pathogenicity of microorganisms, contributing to the destruction of periodontal tissues (gingiva, cement, periodontal ligament, and alveolar bone) and lead to signs and symptoms of periodontitis (gum bleeding, halitosis, clinical attachment loss, tooth mobility, tooth loss) (21, 27). On the other hand, periodontitis also causes an increase in pro-inflammatory cytokines that may increase the systemic inflammatory response and act as a risk factor for the occurrence of anxiety (83, 84). In addition, the behavioral profile of individuals can also influence the maintenance of oral health, through insufficient hygiene added to anxiety related to dental care (85).

In this context, for evaluating periodontitis status, all studies measured PPD and/or CAL (10, 56–65). Other parameters were also used, such as gingival assessment indexes (56–59, 62), bleeding on probing (BOP) (10, 61, 63, 65) and plaque index (56, 57, 59, 60, 63, 65). To define characteristics of the case group (periodontitis) and control group (without periodontitis), anxiety levels were evaluated by psychometric tools composed of self-reported questionnaires such as STAI, BAI, Corah's DAS, SAS, and SCL- 90.

Three studies used STAI method to assess trait and state anxiety, and it was possible to identify a significant correlation between CAL and PPD with higher anxiety scores (56, 59, 65). These studies presented good methodological quality according to the Newcastle-Ottawa protocol. Still, the survey by Vettore et al. (56) showed some gaps in the sample selection domain, where neither the origin of the control group was not described nor the history of the disease. Likewise, Vettore et al. (59) did not declare the subjects' absence of disease history.

The studies by Castro et al. (10) and Solis et al. (57) also assessed anxiety using STAI, but the results showed no significant association between periodontitis and anxiety. In an attempt to understand this outcome, Castro et al. (10) supposed that periodontitis is more directly associated with the subject's demographic and socio-cultural aspects than a direct relationship with psychosocial elements. Meanwhile, Solis et al. (57) considered the hypothesis of individuals' emotional instability when applying the self-reported questionnaire as one of the hypotheses for the absence of an association between periodontitis and anxiety, since the answers depend on the subjective understanding of the emotions themselves.

Pal et al. (64) found an association between anxiety and periodontitis severity, while Castro et al. (10) did not observe this association in the multivariate analysis. It can be suggested that the sample size of the study by Pal et al. (64) contributed to the association outcome (*n* = 300), in contrast to the study by Castro et al. (10), which analyzed only 165 subjects. Also, Castro et al. (10) study a control group with considerably fewer individuals than the case group.

Saletu et al. (58) showed higher anxiety scores in the group of subjects with periodontitis and, in the partial correlation analysis, it was observed that the higher the SAS score, the higher the level of CAL and severity of periodontitis. This study highlighted the increase in cortisol as one of the main factors responsible for emotional changes, such as depression and anxiety which, negatively interfere in the subjects' attitude related to oral health (58). Li et al. (61) also used SAS to assess subjects and found that anxiety levels were higher in the group with periodontitis. They even suggested that psychological treatment may be a protection mechanism against periodontitis progression, along with a frequent oral hygiene habit.

Levin et al. (63) evaluated dental anxiety levels through Corah's DAS (73). They observed that the levels of dental anxiety were higher in subjects with periodontitis, who were more likely to fear the noise of dental instruments and the application of anesthesia (63). However, the study itself points out the limitation in the generalization of the results due to the sample being composed of volunteers from several clinics. Although this may constitute a selection bias, in the analysis of methodological quality according to Newcastle-Ottawa (29), there were no severe risks of bias in the sample selection domain since all individuals in the case and control group were evaluated according to periodontal parameters for the diagnosis of periodontitis. Thus, the subjects were allocated to the respective groups to carry out the anxiety assessment and it was possible to maintain the comparability of the results obtained (63).

In the study by Ababneh et al. (60), although the results showed higher levels of anxiety in the group subjects with aggressive periodontitis, we did not consider these data in this systematic review, because the group with aggressive periodontitis consisted mostly of adolescents. In the analysis of the risk of bias, there was a problem in the definition of control concerning the lack of description about the history of the disease.

Among the studies included in this review, only the study by Laforgia et al. (62) presented more methodological problems than the other studies according to the Newcastle-Ottawa protocol. In the comparability domain, it was observed that the control of confounding factors was not performed. Therefore, although the study concluded an association between periodontitis and anxiety, the outcome may have resulted from the interaction of other factors, as an example, age and sex (62).

All studies evaluated according to the Newcastle-Ottawa protocol did not describe the rate of non-responders (10, 56–65), and cross-sectional studies did not describe how the sample size was defined (57, 58, 62, 64, 65). These gaps do not directly affect the methodological quality of the studies, but they constitute limitations to be considered when carrying out new research.

The level of evidence assessed by the GRADE tool was low for three case-control studies that adopted STAI as a psychometric scale, with no risk of bias based on the assessed domains (10, 56, 59). Vettore et al. (56, 59) showed an association between anxiety and periodontitis; however, the study by Castro (10) did not report a significant association. For the two cross-sectional studies, very low evidence was observed due to inconsistency and inaccuracy of the studies (57, 65), although the outcomes were considered essential for influencing patients' quality of life.

All included studies have general limitations, such as small sample size and the use of self-reported questionnaires to diagnose anxiety, which may generate results with significant variability according to the momentary perception of each participant. In addition, the studies that identified an association between periodontitis and anxiety did not investigate the mechanisms involved in this process. Therefore, the evidence found in this review indicates an association that still needs to be further explored to identify these pathways at the systemic level. In the existent body of evidence retrieved in this review, even though the association was demonstrated, the differences in approaches and possible explanations for the associations prevent more in-depth conclusions in this sense.

## Conclusion

Eight of 11 studies qualifying for inclusion reported higher anxiety levels in subjects with periodontitis, compared to healthy controls, consistent with positive association between periodontitis and anxiety, although with very low certainty of evidence.

## Data Availability Statement

The original contributions generated for this study are included in the article/Supplementary Material, further inquiries can be directed to the corresponding author/s.

## Author Contributions

WA, DS-M, and RL: study concept and design. WA, DS-M, LR, DF, YN, and RL: analysis and interpretation of data. WA, RF, NF, LM, MS, and RL: preparation of the manuscript. LM, CR, MS, and RL: critical revision of the manuscript. All authors contributed to the article and approved the submitted version.

## Conflict of Interest

The authors declare that the research was conducted in the absence of any commercial or financial relationships that could be construed as a potential conflict of interest.

## Publisher's Note

All claims expressed in this article are solely those of the authors and do not necessarily represent those of their affiliated organizations, or those of the publisher, the editors and the reviewers. Any product that may be evaluated in this article, or claim that may be made by its manufacturer, is not guaranteed or endorsed by the publisher.
