# Supplementary material for: Is There Any Association Between Chronic Periodontitis and Anxiety in Adults? A Systematic Review
Source: Front Psychiatry. 2021 Aug 3;12:710606. doi: 10.3389/fpsyt.2021.710606 (PMC8368723; doi:10.3389/fpsyt.2021.710606)
Supplement: Supplementary file 1 [file Table_1.DOCX]

# Supplementary Table 1. Database and Search Strategy

|  |  | | |  |
| --- | --- | --- | --- | --- |
| **DATABASE** | **SEARCH FORMAT** | | |  |
| **PUBMED** | #1  (((((((((Humans[MeSH Terms]) OR humans[Title/Abstract]) OR human[Title/Abstract]) OR Man, Modern[Title/Abstract]) OR Modern Man[Title/Abstract]) OR Man (Taxonomy)[Title/Abstract]) OR Homo sapiens[Title/Abstract]) OR Adult[MeSH Terms]) OR Adult[Title/Abstract]) OR adults[Title/Abstract] | #2  (((((((((((((((((((((((((((((((((((((((((((((((((((((((((((((((((((((((((((((((((((Chronic Periodontitis[MeSH Terms]) OR Chronic Periodontitis[Title/Abstract]) OR Chronic Periodontitides[Title/Abstract]) OR Periodontitides, Chronic[Title/Abstract]) OR Periodontitis, Chronic[Title/Abstract]) OR Adult Periodontitis[Title/Abstract]) OR Adult Periodontitides[Title/Abstract]) OR Periodontitides, Adult[Title/Abstract]) OR Periodontitis, Adult[Title/Abstract]) OR tooth[MeSH Terms]) OR tooth[Title/Abstract]) OR teeth[Title/Abstract]) OR bone loss[MeSH Terms]) OR bone loss[Title/Abstract]) OR Alveolar Bone Losses[Title/Abstract]) OR Alveolar Process Atrophy[Title/Abstract]) OR Alveolar Process Atrophies[Title/Abstract]) OR Alveolar Resorption[Title/Abstract]) OR Alveolar Resorptions[Title/Abstract]) OR Resorption, Alveolar[Title/Abstract]) OR Resorptions, Alveolar[Title/Abstract]) OR Bone Losses, Periodontal[Title/Abstract]) OR Bone Loss, Periodontal[Title/Abstract]) OR Periodontal Bone Losses[Title/Abstract]) OR Periodontal Bone Loss[Title/Abstract]) OR Periodontal Resorption[Title/Abstract]) OR Periodontal Resorptions[Title/Abstract]) OR Resorption, Periodontal[Title/Abstract]) OR Alveolar Bone Atrophy[Title/Abstract]) OR Alveolar Bone Atrophies[Title/Abstract]) OR Bone Atrophies, Alveolar[Title/Abstract]) OR Bone Atrophy, Alveolar[Title/Abstract]) OR Bone Loss, Alveolar[Title/Abstract]) OR Alveolar Process[MeSH Terms]) OR Alveolar Process[Title/Abstract]) OR Alveolar Processes[Title/Abstract]) OR Process, Alveolar[Title/Abstract]) OR Processes, Alveolar[Title/Abstract]) OR Alveolar Ridge[Title/Abstract]) OR Ridge, Alveolar[Title/Abstract]) OR Oral Health[MeSH Terms]) OR Oral Health[Title/Abstract]) OR Health, Oral[Title/Abstract]) OR Oral Medicine[MeSH Terms]) OR Oral Medicine[Title/Abstract]) OR Stomatology[Title/Abstract]) OR Medicine, Oral[Title/Abstract]) OR Periodontium[MeSH Terms]) OR Periodontium[Title/Abstract]) OR Periodontiums[Title/Abstract]) OR Tooth Supporting Structures[Title/Abstract]) OR Structure, Tooth Supporting[Title/Abstract]) OR Structures, Tooth Supporting[Title/Abstract]) OR Supporting Structure, Tooth[Title/Abstract]) OR Supporting Structures, Tooth[Title/Abstract]) OR Tooth Supporting Structure[Title/Abstract]) OR Parodontium[Title/Abstract]) OR Parodontiums[Title/Abstract]) OR Paradentium[Title/Abstract]) OR Paradentiums[Title/Abstract]) OR Gingiva[MeSH Terms]) OR Gingiva[Title/Abstract]) OR Gums[Title/Abstract]) OR Gum[Title/Abstract]) OR Interdental Papilla[Title/Abstract]) OR Papilla, Interdental[Title/Abstract]) OR Periodontal Index[MeSH Terms]) OR Periodontal Index[Title/Abstract]) OR Index, Periodontal[Title/Abstract]) OR Indices, Periodontal[Title/Abstract]) OR Periodontal Indices[Title/Abstract]) OR Periodontal Indexes[Title/Abstract]) OR Indexes, Periodontal[Title/Abstract]) OR Community Periodontal Index of Treatment Needs[Title/Abstract]) OR CPITN[Title/Abstract]) OR Bleeding on Probing, Gingival[Title/Abstract]) OR Gingival Bleeding on Probing[Title/Abstract]) OR Gingival Index[Title/Abstract]) OR Gingival Indices[Title/Abstract]) OR Index, Gingival[Title/Abstract]) OR Indices, Gingival[Title/Abstract]) OR Gingival Indexes[Title/Abstract]) OR Indexes, Gingival[Title/Abstract]) OR Periodontal status[Title/Abstract] | #3  ((((((((((((((((((((((((((((((((((((Anxiety[MeSH Terms]) OR Anxiety[Title/Abstract]) OR Hypervigilance[Title/Abstract]) OR Nervousness[Title/Abstract]) OR Social Anxiety[Title/Abstract]) OR Anxieties, Social[Title/Abstract]) OR Anxiety, Social[Title/Abstract]) OR Social Anxieties[Title/Abstract]) OR Anxiety Disorders[MeSH Terms]) OR Anxiety Disorders[Title/Abstract]) OR Anxiety Disorder[Title/Abstract]) OR Disorder, Anxiety[Title/Abstract]) OR Disorders, Anxiety[Title/Abstract]) OR Neuroses, Anxiety[Title/Abstract]) OR Anxiety Neuroses[Title/Abstract]) OR Anxiety States, Neurotic[Title/Abstract]) OR Anxiety State, Neurotic[Title/Abstract]) OR Neurotic Anxiety State[Title/Abstract]) OR Neurotic Anxiety States[Title/Abstract]) OR State, Neurotic Anxiety[Title/Abstract]) OR States, Neurotic Anxiety[Title/Abstract]) OR Anxiety, Separation[MeSH Terms]) OR Anxiety, Separation[Title/Abstract]) OR Separation Anxiety[Title/Abstract]) OR Separation Anxiety Disorder[Title/Abstract]) OR Anxiety Disorder, Separation[Title/Abstract]) OR Affective Symptoms[MeSH Terms]) OR Affective Symptoms[Title/Abstract]) OR Affective Symptom[Title/Abstract]) OR Symptom, Affective[Title/Abstract]) OR Symptoms, Affective[Title/Abstract]) OR Emotional Disturbances[Title/Abstract]) OR Disturbances, Emotional[Title/Abstract]) OR Disturbance, Emotional[Title/Abstract]) OR Emotional Disturbance[Title/Abstract] |  |
|  | **Final search #1 AND #2 AND #3** | | | |
| **SCOPUS** | #1  TITLE-ABS-KEY (Human*) OR TITLE-ABS-KEY (“Man, Modern”) OR TITLE-ABS-KEY (“Modern Man”) OR TITLE-ABS-KEY (“Man (Taxonomy)”) OR TITLE-ABS-KEY (“Homo sapiens”) OR TITLE-ABS-KEY (Adult*) | #2  TITLE-ABS-KEY ("Chronic Periodontiti*") OR TITLE-ABS-KEY ("Periodontitides, Chronic") OR TITLE-ABS-KEY ("Periodontitis, Chronic") OR TITLE-ABS-KEY ("Adult Periodontiti*") OR TITLE-ABS-KEY ("Periodontitides, Adult") OR TITLE-ABS-KEY ("Periodontitis, Adult") OR TITLE-ABS-KEY (tooth) OR TITLE-ABS-KEY (teeth) OR TITLE-ABS-KEY ("bone loss") OR TITLE-ABS-KEY ("Alveolar Bone Losses") OR TITLE-ABS-KEY ("Alveolar Process Atroph*") OR TITLE-ABS-KEY ("Alveolar Resorption*") OR TITLE-ABS-KEY ("Resorption, Alveolar") OR TITLE-ABS-KEY ("Resorptions, Alveolar") OR TITLE-ABS-KEY ("Bone Loss, Periodontal") OR TITLE-ABS-KEY ("Bone Losses, Periodontal") OR TITLE-ABS-KEY ("Periodontal Bone Loss*") OR TITLE-ABS-KEY ("Periodontal Resorption*") OR TITLE-ABS-KEY ("Resorption, Periodontal") OR TITLE-ABS-KEY ("Alveolar Bone Atroph*") OR TITLE-ABS-KEY ("Bone Atrophies, Alveolar") OR TITLE-ABS-KEY ("Bone Atrophy, Alveolar") OR TITLE-ABS-KEY ("Bone Loss, Alveolar") OR TITLE-ABS-KEY ("Alveolar Process*") OR TITLE-ABS-KEY ("Process, Alveolar") OR TITLE-ABS-KEY ("Processes, Alveolar") OR TITLE-ABS-KEY ("Alveolar Ridge") OR TITLE-ABS-KEY ("Ridge, Alveolar") OR TITLE-ABS-KEY ("Oral Health") OR TITLE-ABS-KEY ("Health, Oral") OR TITLE-ABS-KEY ("Oral Medicine") OR TITLE-ABS-KEY (stomatology) OR TITLE-ABS-KEY ("Medicine, Oral") OR TITLE-ABS-KEY (periodontium*) OR TITLE-ABS-KEY ("Tooth Supporting Structures") OR TITLE-ABS-KEY ("Structure, Tooth Supporting") OR TITLE-ABS-KEY ("Structures, Tooth Supporting") OR TITLE-ABS-KEY ("Supporting Structure, Tooth") OR TITLE-ABS-KEY ("Supporting Structures, Tooth") OR TITLE-ABS-KEY ("Tooth Supporting Structure") OR TITLE-ABS-KEY (parodontium*) OR TITLE-ABS-KEY (paradentium*) OR TITLE-ABS-KEY (gingiva) OR TITLE-ABS-KEY (gum*) OR TITLE-ABS-KEY ("Interdental Papilla") OR TITLE-ABS-KEY ("Papilla, Interdental") OR TITLE-ABS-KEY ("Periodontal Index") OR TITLE-ABS-KEY ("Index, Periodontal") OR TITLE-ABS-KEY ("Indices, Periodontal") OR TITLE-ABS-KEY ("Periodontal Indices") OR TITLE-ABS-KEY ("Periodontal Indexes") OR TITLE-ABS-KEY ("Indexes, Periodontal") OR TITLE-ABS-KEY ("Community Periodontal Index of Treatment Needs") OR TITLE-ABS-KEY (cpitn) OR TITLE-ABS-KEY ("Bleeding on Probing, Gingival") OR TITLE-ABS-KEY ("Gingival Bleeding on Probing") OR TITLE-ABS-KEY ("Gingival Index") OR TITLE-ABS-KEY ("Gingival Indices") OR TITLE-ABS-KEY ("Index, Gingival") OR TITLE-ABS-KEY ("Indices, Gingival") OR TITLE-ABS-KEY ("Gingival Indexes") OR TITLE-ABS-KEY ("Indexes, Gingival") OR TITLE-ABS-KEY ("Periodontal status") | #3  TITLE-ABS-KEY (Anxiety) OR TITLE-ABS-KEY (Hypervigilance) OR TITLE-ABS-KEY (Nervousness) OR TITLE-ABS-KEY (“Social Anxiety”) OR TITLE-ABS-KEY (“Anxieties, Social”) OR TITLE-ABS-KEY (“Anxiety, Social”) OR TITLE-ABS-KEY (“Anxiety Disorder*”) OR TITLE-ABS-KEY (“Disorder, Anxiety”) OR TITLE-ABS-KEY (“Disorders, Anxiety”) OR TITLE-ABS-KEY (“Neuroses, Anxiety”) OR TITLE-ABS-KEY (“Anxiety Neuroses”) OR TITLE-ABS-KEY (“Anxiety States, Neurotic”) OR TITLE-ABS-KEY (“Anxiety State, Neurotic”) OR TITLE-ABS-KEY (“Neurotic Anxiety State*”) OR TITLE-ABS-KEY (“State, Neurotic Anxiety”) OR TITLE-ABS-KEY (“States, Neurotic Anxiety”) OR TITLE-ABS-KEY (“Anxiety, Separation”) OR TITLE-ABS-KEY (“Separation Anxiety”) OR TITLE-ABS-KEY (“Separation Anxiety Disorder”) OR TITLE-ABS-KEY (“Anxiety Disorder, Separation”) OR TITLE-ABS-KEY (“Affective Symptoms”) OR TITLE-ABS-KEY (“Affective Symptom”) OR TITLE-ABS-KEY (“Symptom, Affective”) OR TITLE-ABS-KEY (“Symptoms, Affective”) OR TITLE-ABS-KEY (“Emotional Disturbances”) OR TITLE-ABS-KEY (“Disturbance, Emotional”) OR TITLE-ABS-KEY (“Disturbances, Emotional”) OR TITLE-ABS-KEY (“Emotional Disturbance”) |  |
|  | **Final search #1 AND #2 AND #3** | | |  |
| **WEB OF SCIENCE** | #1  TS=(Human*) OR TS=(“Man, Modern”) OR TS=(“Modern Man”) OR TS=(“Man (Taxonomy)”) OR TS=(“Homo sapiens”) OR TS=(Adult*) | #2  TS=(“Chronic Periodontitis”) OR TS=(“Chronic Periodontitides”) OR TS=(“Periodontitides, Chronic”) OR TS=(“Periodontitis, Chronic”) OR TS=(“Adult Periodontitis”) OR TS=(“Adult Periodontitides”) OR TS=(“Periodontitides, Adult”) OR TS=(“Periodontitis, Adult”) OR TS=(tooth) OR TS=(teeth) OR TS=(“bone loss”) OR TS=(“Alveolar Bone Losses”) OR TS=(“Alveolar Process Atroph*”) OR TS=(“Alveolar Resorption*”) OR TS=(“Resorption, Alveolar”) OR TS=(“Resorptions, Alveolar”) OR TS=(“Bone Loss, Periodontal”) OR TS=(“Bone Losses, Periodontal”) OR TS=(“Periodontal Bone Loss*”) OR TS=(“Periodontal Resorption*”) OR TS=(“Resorption, Periodontal”) OR TS=(“Alveolar Bone Atroph*”) OR TS=(“Bone Atrophies, Alveolar”) OR TS=(“Bone Atrophy, Alveolar”) OR TS=(“Bone Loss, Alveolar”) OR TS=(“Alveolar Process*”) OR TS=(“Process, Alveolar”) OR TS=(“Processes, Alveolar”) OR TS=(“Alveolar Ridge”) OR TS=(“Ridge, Alveolar”) OR TS=(“Oral Health”) OR TS=(“Health, Oral”) OR TS=(“Oral Medicine”) OR TS=(“Stomatology”) OR TS=(“Medicine, Oral”) OR TS=(Periodontium*) OR TS=(“Tooth Supporting Structures”) OR TS=(“Structure, Tooth Supporting”) OR TS=(“Structures, Tooth Supporting”) OR TS=(“Supporting Structure, Tooth”) OR TS=(“Supporting Structures, Tooth”) OR TS=(“Tooth Supporting Structure”) OR TS=(Parodontium*) OR TS=(Paradentium*) OR TS=(Gingiva) OR TS=(Gum*) OR TS=(“Interdental Papilla”) OR TS=(“Papilla, Interdental”) OR TS=(“Periodontal Index”) OR TS=(“Index, Periodontal”) OR TS=(“Indices, Periodontal”) OR TS=(“Periodontal Indices”) OR TS=(“Periodontal Indexes”) OR TS=(“Indexes, Periodontal”) OR TS=(“Community Periodontal Index of Treatment Needs”) OR TS=(CPITN) OR TS=(“Bleeding on Probing, Gingival”) OR TS=(“Gingival Bleeding on Probing”) OR TS=(“Gingival Index”) OR TS=(“Gingival Indices”) OR TS=(“Index, Gingival”) OR TS=(“Indices, Gingival”) OR TS=(“Gingival Indexes”) OR TS=(“Indexes, Gingival”) OR TS=(“Periodontal status”) | #3  TS=(Anxiety) OR TS=(Hypervigilance) OR TS=(Nervousness) OR TS=(“Social Anxiety”) OR TS=(“Anxieties, Social”) OR TS=(“Anxiety, Social”) OR TS=(“Social Anxieties”) OR TS=(“Anxiety Disorder*”) OR TS=(“Disorder, Anxiety”) OR TS=(“Disorders, Anxiety”) OR TS=(“Neuroses, Anxiety”) OR TS=(“Anxiety Neuroses”) OR TS=(“Anxiety States, Neurotic”) OR TS=(“Anxiety State, Neurotic”) OR TS=(“Neurotic Anxiety State*”) OR TS=(“State, Neurotic Anxiety”) OR TS=(“States, Neurotic Anxiety”) OR TS=(“Anxiety, Separation”) OR TS=(“Separation Anxiety”) OR TS=(“Separation Anxiety Disorder”) OR TS=(“Anxiety Disorder, Separation”) OR TS=(“Affective Symptoms”) OR TS=(“Affective Symptom”) OR TS=(“Symptom, Affective”) OR TS=(“Symptoms, Affective”) OR TS=(“Emotional Disturbances”) OR TS=(“Disturbance, Emotional”) OR TS=(“Disturbances, Emotional”) OR TS=(“Emotional Disturbance”) |  |
|  | **Final search #1 AND #2 AND #3** | | |  |
| **COCHRANE** | #1  (Humans OR Human OR “Man, Modern” OR “Modern Man” OR “Man (Taxonomy)” OR “Homo sapiens” OR “Adult” OR “Adults”):ti,ab,kw | #2  (“Chronic Periodontitis” OR “Chronic Periodontitides” OR “Periodontitides, Chronic” OR “Periodontitis, Chronic” OR “Adult Periodontitis” OR “Adult Periodontitides” OR “Periodontitides, Adult” OR “Periodontitis, Adult” OR tooth OR teeth OR “bone loss” OR “Alveolar Bone Losses” OR “Alveolar Process Atrophy” OR “Alveolar Process Atrophies” OR “Alveolar Resorption” OR “Alveolar Resorptions” OR “Resorption, Alveolar” OR “Resorptions, Alveolar” OR “Bone Loss, Periodontal” OR “Bone Losses, Periodontal” OR “Periodontal Bone Losses” OR “Periodontal Bone Loss” OR “Periodontal Resorption” OR “Periodontal Resorptions” OR “Resorption, Periodontal” OR “Alveolar Bone Atrophy” OR “Alveolar Bone Atrophies” OR “Bone Atrophies, Alveolar” OR “Bone Atrophy, Alveolar” OR “Bone Loss, Alveolar” OR “Alveolar Process” OR “Alveolar Processes” OR “Process, Alveolar” OR “Processes, Alveolar” OR “Alveolar Ridge” OR “Ridge, Alveolar” OR “Oral Health” OR “Health, Oral” OR “Oral Medicine” OR Stomatology OR “Medicine, Oral” OR Periodontium OR Periodontiums OR “Tooth Supporting Structures” OR “Structure, Tooth Supporting” OR “Structures, Tooth Supporting” OR “Supporting Structure, Tooth” OR “Supporting Structures, Tooth” OR “Tooth Supporting Structure” OR Parodontium OR Parodontiums OR Paradentium OR Paradentiums OR Gingiva OR Gums OR Gum OR “Interdental Papilla” OR “Papilla, Interdental” OR “Periodontal Index” OR “Index, Periodontal” OR “Indices, Periodontal” OR “Periodontal Indices” OR “Periodontal Indexes” OR “Indexes, Periodontal” OR “Community Periodontal Index of Treatment Needs” OR CPITN OR “Bleeding on Probing, Gingival” OR “Gingival Bleeding on Probing” OR “Gingival Index” OR “Gingival Indices” OR “Index, Gingival” OR “Indices, Gingival” OR “Gingival Indexes” OR “Indexes, Gingival” OR “Periodontal status”):ti,ab,kw | #3  (Anxiety OR Hypervigilance OR Nervousness OR “Social Anxiety” OR “Anxieties, Social” OR “Anxiety, Social” OR “Social Anxieties” OR “Anxiety Disorders” OR “Anxiety Disorder” OR “Disorder, Anxiety” OR “Disorders, Anxiety” OR “Neuroses, Anxiety” OR “Anxiety Neuroses” OR “Anxiety States, Neurotic” OR “Anxiety State, Neurotic” OR “Neurotic Anxiety State” OR “Neurotic Anxiety States” OR “State, Neurotic Anxiety” OR “States, Neurotic Anxiety” OR “Anxiety, Separation” OR “Separation Anxiety” OR “Separation Anxiety Disorder” OR “Anxiety Disorder, Separation” OR “Affective Symptoms” OR “Affective Symptom” OR “Symptom, Affective” OR “Symptoms, Affective” OR “Emotional Disturbances” OR “Disturbance, Emotional” OR “Disturbances, Emotional” OR “Emotional Disturbance”):ti,ab,kw |  |
|  | **Final search #1 AND #2 AND #3** | | |  |
| **LILACS** | #1  (Human$) OR (Man, Modern) OR (Modern Man) OR (Man (Taxonomy)) OR (Homo sapiens) OR (Adult$) | #2  (Chronic Periodontiti$) OR (Periodontitides, Chronic) OR (Periodontitis, Chronic) OR (Adult Periodontiti$) OR (Periodontitides, Adult) OR (Periodontitis, Adult) OR (tooth) OR (teeth) OR (bone loss) OR (Alveolar Bone Losses) OR (Alveolar Process Atroph$) OR (Alveolar Resorption$) OR (Resorption, Alveolar) OR (Resorptions, Alveolar) OR (Bone Loss, Periodontal) OR (Bone Losses, Periodontal) OR (Periodontal Bone Loss$) OR (Periodontal Resorption$) OR (Resorption, Periodontal) OR (Alveolar Bone Atroph$) OR (Bone Atrophies, Alveolar) OR (Bone Atrophy, Alveolar) OR (Bone Loss, Alveolar) OR (Alveolar Process$) OR (Process, Alveolar) OR (Processes, Alveolar) OR (Alveolar Ridge) OR (Ridge, Alveolar) OR (Oral Health) OR (Health, Oral) OR (Oral Medicine) OR (Stomatology) OR (Medicine, Oral) OR (Periodontium$) OR (Tooth Supporting Structures) OR (Structure, Tooth Supporting) OR (Structures, Tooth Supporting) OR (Supporting Structure, Tooth) OR (Supporting Structures, Tooth) OR (Tooth Supporting Structure) OR (Parodontium$) OR (Paradentium$) OR (Gingiva) OR (Gum$) OR (Interdental Papilla) OR (Papilla, Interdental) OR (Periodontal Index) OR (Index, Periodontal) OR (Indices, Periodontal) OR (Periodontal Indices) OR (Periodontal Indexes) OR (Indexes, Periodontal) OR (Community Periodontal Index of Treatment Needs) OR (CPITN) OR (Bleeding on Probing, Gingival) OR (Gingival Bleeding on Probing) OR (Gingival Index) OR (Gingival Indices) OR (Index, Gingival) OR (Indices, Gingival) OR (Gingival Indexes) OR (Indexes, Gingival) OR (Periodontal status) | #3  (Anxiety) OR (Hypervigilance) OR (Nervousness) OR (Social Anxiety) OR (Anxieties, Social) OR (Anxiety, Social) OR (Social Anxieties) OR (Anxiety Disorder$) OR (Disorder, Anxiety) OR (Disorders, Anxiety) OR (Neuroses, Anxiety) OR (Anxiety Neuroses) OR (Anxiety States, Neurotic) OR (Anxiety State, Neurotic) OR (Neurotic Anxiety State$) OR (State, Neurotic Anxiety) OR (States, Neurotic Anxiety) OR (Anxiety, Separation) OR (Separation Anxiety) OR (Separation Anxiety Disorder) OR (Anxiety Disorder, Separation) OR (Affective Symptom$) OR (Symptom, Affective) OR (Symptoms, Affective) OR (Emotional Disturbances) OR (Disturbance, Emotional) OR (Disturbances, Emotional) OR (Emotional Disturbance) |  |
|  | **Final search #1 AND #2 AND #3** | | |  |
| **OPENGREY** | Humans AND anxiety AND periodontitis | | |  |
| **GREY SOURCE** | Humans AND anxiety AND periodontitis | | |  |

**Supplementary Table 2.** Evaluation of the methodological quality of studies according to the Newcastle-Ottawa protocol for case-control studies.

| **Criterion** | **Checklist** | **Description** |
| --- | --- | --- |
| **Selection** | Is the Case Definition Adequate? | 1. Requires some independent validation (e.g. >1 person/record/time/process to extract information, or reference to primary record source such as x-rays or medical/hospital records): * 2. Record linkage (e.g. ICD codes in database) or self-report with no reference to primary record 3. No description |
|  | Representativeness of the Cases | 1. All eligible cases with outcome of interest over a defined period of time, all cases in a defined catchment area, all cases in a defined hospital or clinic, group of hospitals, health maintenance organisation, or an appropriate sample of those cases (e.g. random sample): * 2. Not satisfying requirements in part (a), or not stated. |
|  | Selection of Controls | This item assesses whether the control series used in the study is derived from the same population as the cases and essentially would have been cases had the outcome been present.   1. Community controls (i.e. same community as cases and would be cases if had outcome): * 2. Hospital controls, within same community as cases (i.e. not another city) but derived from a hospitalized population 3. No description |
|  | Definition of Controls | 1. If cases are first occurrence of outcome, then it must explicitly state that controls have no history of this outcome. If cases have new (not necessarily first) occurrence of outcome, then controls with previous occurrences of outcome of interest should not be excluded. * 2. No mention of history of outcome |
| **Comparability**  (Maximum 2 stars) | Comparability of Cases and Controls on the Basis of the Design or Analysis | A maximum of 2 stars (**) can be allotted in this category.  Either cases and controls must be matched in the design and/or confounders must be adjusted for in the analysis. Statements of no differences between groups or that differences were not statistically significant are not sufficient for establishing comparability. Note: If the odds ratio for the exposure of interest is adjusted for the confounders listed, then the groups will be considered to be comparable on each variable used in the adjustment.  There may be multiple ratings for this item for different categories of exposure (e.g. ever vs. never, current vs. previous or never)  Age: *  Other controlled factors: * |
| **Exposure** | Ascertainment of Exposure | 1. secure record (e.g. surgical records) * 2. structured interview where blind to case/control status * 3. interview not blinded to case/control status 4. written self-report or medical record only 5. no description |
|  | Same method of ascertainment for cases and controls | 1. yes * 2. no |
|  | Non-Response Rate | 1. same rate for both groups * 2. non respondents described 3. rate different and no designation |

**Supplementary Table 3.** Evaluation of the methodological quality of studies according to the Newcastle-Ottawa protocol adapted for cross-sectional studies.

| **Criterion** | **Checklist** | **Description** |
| --- | --- | --- |
| **Selection**  (Maximum 5 stars) | Representativeness of the sample | 1. Truly representative of the average in the target population. * (all subjects or random sampling) 2. Somewhat representative of the average in the target population. * (non- random sampling) 3. Selected group of users. 4. No description of the sampling strategy. |
|  | Sample size | 1. Justified and satisfactory   *   1. Not justified |
|  | Non-respondents | 1. Comparability between respondents and non-respondents characteristics is established, and the response rate is satisfactory. * 2. The response rate is unsatisfactory, or the comparability between respondents and non-respondents is unsatisfactory. 3. No description of the response rate or the characteristics of the responders and the non-responders. |
|  | Ascertainment of the exposure (risk factor) | 1. Validated measurement tool. ** 2. Non-validated measurement tool, but the tool is available or described. * 3. No description of the measurement tool |
| **Comparability**  (Maximum 2 stars) | The subjects in different outcome groups are comparable, based on the study design or analysis. Confounding factors are controlled. | 1. The study controls for the most important factor (select one). * 2. The study control for any additional factor. * |
| **Outcome** (Maximum 3 stars) | Assessment of the outcome | 1. Independent blind assessment. ** 2. Record linkage. ** 3. Self-report. * 4. No description |
|  | Statistical test | 1. The statistical test used to analyze the data is clearly described and appropriate, and the measurement of the association is presented, including confidence intervals and the probability level (p value). * 2. The statistical test is not appropriate, not described or incomplete |
